# Self-reporting of hearing loss and tinnitus in the diagnosis of ototoxicity by meglumine antimoniate in patients treated for American tegumentary Leishmaniasis

**DOI:** 10.1371/journal.pone.0296728

**Published:** 2024-02-14

**Authors:** Claudia Cristina Jardim Duarte, Tania Salgado de Sousa Torraca, Débora de Oliveira Bezerra, Renata de Barcelos Oliveira, Nayany Kerollyany Sousa Leite, Raquel de Vasconcellos Carvalhaes de Oliveira, Maria Helena Araújo-Melo, Maria Inês Fernandes Pimentel, Ananda Dutra da Costa, Érica de Camargo Ferreira Vasconcellos, Marcelo Rosandiski Lyra, Ester Cleisla dos Anjos Soares, Lúcia Regina do Nascimento Brahim Paes, Mariza Mattos Salgueiro, Armando de Oliveira Schubach, Cláudia Maria Valete

**Affiliations:** 1 Evandro Chagas National Institute of Infectious Disease, Oswaldo Cruz Foundation, Rio de Janeiro, Brazil; 2 Department of Otorhinolaryngology and Ophtalmology, Federal University of Rio de Janeiro, Rio de Janeiro, Brazil; 3 Brazil´s navy, Rio de Janeiro, Brazil; 4 Department of Otorhinolaringology, Federal University of the Estate of Rio de Janeiro, Rio de Janeiro, Brazil; 5 Medicine School, Souza Marques Technical and Scientific Foundation, Rio de Janeiro, Brazil; 6 Productivity fellowship holder of the Brazilian National Council for Scientific and Technological Development (CNPQ), Brasília, Brazil; Universidade Federal da Bahia, BRAZIL

## Abstract

**Introduction:**

American Tegumentary Leishmaniasis (ATL) treatment is based on pentavalent antimonials (Sb^5+^), but these drugs have been associated to several adverse effects. Hearing loss and tinnitus during treatment with meglumine antimoniate (MA) have already been reported. This study aimed to describe the usefulness of self-reporting of hearing loss and tinnitus in diagnosing MA-induced ototoxicity.

**Methods:**

A prospective longitudinal study was conducted with 102 patients with parasitological diagnosis of ATL, treated with different MA schemes. The presence of clinical auditory toxicity was defined as the emergence or worsening of self-reporting hearing loss and/or tinnitus during monitoring. Measures of sensitivity, specificity, and the positive and negative predictive value of the patient’s self-reporting of hearing loss and tinnitus in relation to the result of the audiometric test (considered the gold standard) were calculated.

**Results:**

The age of the evaluated patients ranged from 15 to 81 years, with a median of 41 years, and most were male (73.5%). Seventy-five patients (73.5%) had cutaneous leishmaniasis and 27 (26.5%) mucosal leishmaniasis. Eighty-six patients (84.3%) received intramuscular (IM) treatment and 16 (15.7%) were treated with intralesional MA. During treatment, 18 (17,6%) had tinnitus and 7 (6,9%) had complaint of hearing loss. 53 (52%) patients had cochlear toxicity confirmed by tone threshold audiometry and high frequency audiometry, from which 60% received a dose of 20 mg Sb^5+^/kg/day (p = 0.015) and 96.2% were treated with IM MA (p = 0.001). Tinnitus has greater specificity and positive predictive value than hearing loss, with a low number of false positives, but with a high false negative value.

**Conclusion:**

Although the large number of false negatives suggests that self-report of hearing loss or tinnitus cannot be considered a good screening test for referring the patient to an audiometry, the low number of false positives suggests the need to value the patient’s complaint for referral. Otherwise, this study reinforces the importance of audiological monitoring during treatment with MA, especially in those patients with self-reporting of hearing loss or tinnitus when treated with 20 mg Sb^5+^/kg/day via IM.

## Introduction

American tegumentary Leishmaniasis (ATL) is a non-contagious infectious disease caused by different types of protozoa of the *Leishmania* genus that affects skin (cutaneous leishmaniasis–CL) and mucous membranes (mucosal leishmaniasis–ML) [1]).

Antimoniate (MA) (Glucantime ®) has been the most used ATL treatment, particularly in Brazil [1]. World Health Organization [2] recommends ATL treatment with 20 mg Sb^5+^/kg/day intramuscular (IM) during 20 days for CL and during 30 days for ML with no limits for maximum daily dose. Brazilian Ministry of Health recommends the same dose as but limited to a maximum daily dose of 1,215 mg Sb^5+^, which is equivalent to 3 MA vials or 15 mL [1]. An alternative treatment plan for ATL is the use of 5 mg Sb^5+^/kg/day IM during 30 days for CL, and until mucosal lesion healing, with a maximum of 120 doses for ML [1,3]. Alternatively, CL may also be treated with up to three intralesional (IL) antimoniate (via subcutaneous) injections with good therapeutic results [1]. The most common adverse effects of MA include general symptoms as myalgia and arthralgia, elevated serum urea, creatinine, aspartate aminotransferase, alanine aminotransferase, amylase and lipase, and electrocardiographic alterations, being more frequent and more severe in the elderly and patients with comorbidities [1,4].

Ototoxicity is described as damage of the cochlea and/or vestibular apparatus caused by the exposure to chemical substances. Particularly, cochlear toxicity refers to damage affecting the auditory system resulting in sensorineural hearing loss and/or tinnitus. The use of ototoxic drugs is common, but the evaluation of the auditory function with adequate exams is not frequently available in clinical practice in primary health units [5]. The gold standard test for cochlear toxicity diagnosis is tone threshold audiometry (TTA) and high frequency audiometry (HFA) (American Speech Hearing Association, [6]. It has been described that self-reporting is related to moderate hearing losses and that audiometric evidence is needed for establishing the diagnosis [7].

Dizziness during ML treatment with MA was previously reported [8]. Our group reported the first proven case of cochleovestibular ototoxicity in an elderly ML patient being treated with 20 mg Sb^5+^/kg/day MA [9], and subsequently changes in otoacoustic emissions associated with ototoxicity due to the use of MA were demonstrated [10]. The aim of this study is to describe the usefulness of self-reporting of hearing loss and tinnitus in diagnosing MA-induced ototoxicity.

## Methods

This is an observative prospective and longitudinal study with patients, convenience sample older than 15 years with parasitological confirmed diagnosis of ATL by one or more methods (direct examination ‐ skin smear or imprint, histopathology, culture, immunohistochemistry, or polymerase chain reaction), and treated with MA, at Evandro Chagas National Institute of Infectious Diseases (INI), Oswaldo Cruz Foundation, Rio de Janeiro, Brazil, from 2008 to 2015.

The study was approved by the Ethics Committee of the INI (approval number 3.420.434) and all the patients agreed to participate in this study and signed a free informed consent form.

Before, during, and at the end of treatment, and within one and two months after the end of treatment, patients answered a questionnaire with information about hearing loss and tinnitus, underwent otoscopy, tone threshold audiometry (TTA) (0.25 KHz to 8 KHz) and high frequency audiometry (HFA) (9 KHz to 16 KHz) (MADSEN ITERA II audiometer). All the examinations were conducted in an acoustic booth. Auditory thresholds above 25 dB in TTA and HFA were considered as hearing loss. Patients who had not answered to the standardized questionnaire or had not performed tone threshold audiometry (TTA) and high-frequency audiometry (HFA) pre-treatment, during, and after treatment were excluded. Patients with diseases such as Alzheimer´s and other mental disabilities that could affect their response to the questionnaire were also excluded. Sex, age, clinical form of ATL and treatment scheme of MA were collected from the medical record.

The presence of clinical auditory toxicity was defined as the emergence or worsening of self-reporting hearing loss and/or tinnitus during monitoring. The results of the clinical complaints were interpreted by the audiology team without knowledge of the audiometry results and previous questionnaires. The gold standard test for cochlear toxicity diagnosis is tone threshold audiometry (TTA) and high frequency audiometry (HFA). Criteria to define cochlear toxicity are: (a) • 20 dB decrease at any one test frequency, (b) • 10 dB decrease at any two adjacent test frequencies, or (c) loss of response at three consecutive test frequencies where responses were previously obtained (the third criterion refers specifically to the highest frequencies tested, where earlier responses are obtained close to the limits of audiometric output and later responses cannot be obtained at the limits of the audiometer) [6]. There were no uninterpretable test results. To find the best accuracy of complaints of hearing loss and tinnitus in relation to the frequencies with alterations according to this criteria [6], we evaluated three different frequency ranges: 1–0.25 KHz to 16 KHz. 2–0.25 KHz to 8 KHz; 3- frequencies of 0,5 KHz, 1 KHz, 2 KHz and 4 KHz.

Data was analyzed in the software R version 4.1.2 (R Core Team, 2021). The normality of the age was rejected by the Shapiro-Wilk test. Proportions were calculated for the categorical variables (sex, clinical form of ATL and treatment scheme of MA) and the summary measures (median, minimum and maximum) for the age. Measures of sensitivity, specificity, and the positive predictive value (PPV) and negative predictive value (NPV) of the patient’s complaints in relation to the result of the audiometric test (considered the gold standard) were calculated. Accuracy was stratified according to age to assess possible confusing effects.

The study was approved by the Committee on Ethics in Research of INI ‐ FIOCRUZ, Rio de Janeiro, Brazil. All patients signed a free and informed consent form. In the case of the minors/children enrolled in our study, we obtained written informed consent from the guardians on behalf of them.

## Results

Of the 123 patients, 20 were excluded. A total of 102 patients with ATL treated with MA were included and evaluated in the study, 75 (73.5%) with CL and 27 (26.5%) with ML. Eighty-six patients (84.3%) received IM treatment and 16 (15.7%) were treated with IL MA. The age ranged from 15 to 81 years old (median of 41 years old), most being male (73.5%). During treatment, it was observed that 53 (52%) patients had cochlear toxicity confirmed by tone threshold audiometry and high frequency audiometry, from which 60% were treated with a MA dose of 20 mg Sb^5+^/kg/day (p = 0.015) and 96.2% were treated with IM MA (p = 0.001). Twenty eight patients (27,5%) did not have ototoxicity reversal after the end of treatment. During the auditory monitoring, seven patients (6.9%) reported onset or worsening of only hearing loss, 18 patients (17.6%) of only tinnitus, and 23 patients (22.5%) of both hearing loss and tinnitus. The measures of sensitivity, specificity, positive (PPV) and negative (NPV) predictive values, false positive and false negative of self-reported hearing loss, of self-reported tinnitus and of self-reported hearing loss or tinnitus in relation to the three different frequencies ranges are, respectively, at the Tables 1–3.

**Table 1:** Sensitivity, specificity, predictive values, false positives and false negatives in the diagnosis of cochlear toxicity from self-reported hearing loss in relation to the ototoxicity alteration at three different frequencies ranges of tone threshold audiometry in 102 patients with American Tegumentary Leishmaniasis treated with meglumine antimoniate. Evandro Chagas National Institute of Infectious Diseases, Oswaldo Cruz Foundation, Rio de Janeiro, Brazil.

FR	Category	Sensitivity % (CI)	Specificity % (CI)	PPV % (CI)	NPV % (CI)	FP % (CI)	FN % (CI)
0.25–16 KHz	Total	9 (3–21)	96 (86–100)	71 (29–96)	49 (39–60)	4 (0–14)	91(79–97)
	Up to 59 years	5 (1–17)	97 (87–100)	67 (9–99)	50 (38–62)	3 (0–13)	95 (83–99)
	≥ 60 years	25 (5–57)	89 (52–100)	75 (19–99)	47 (23–72)	11 (0–48)	75 (43–95)
0.25–16 KHz	Total	15 (4–35)	96 (81–100)	80 (28–99)	54 (39–69)	4 (0–19)	85 (65–96)
	Up to 59 years	11 (1–35)	100 (85–100)	100 (16–100)	59 (42–74)	0 (0–15)	89 (65–99)
	≥ 60 years	25 (3–65)	75 (19–99)	67 (9–99)	33 (7–70)	25 (1–81)	75 (35–97)
0.5, 1, 2, 4 KHz	Total	17 (4–41)	94 (81–99)	60 (15–95)	69 (54–81)	6 (1–19)	83 (59–96)
	Up to 59 years	13 (2–40)	100 (87–100)	100 (16–100)	67 (50–81)	0 (0–13)	87 (60–98)
	≥ 60 years	33 (1–91)	78 (40–97)	33 (1–91)	78 (40–97)	22 (3–60)	67 (9–99)

FR = frequencies range; PPV = positive predictive value; NPV = negative predictive value; FP = false positive; FN = false negative.

**Table 2:** Sensitivity, specificity, predictive values, false positives and false negatives in the diagnosis of cochlear toxicity from self-reported tinnitus in relation to the ototoxicity alteration at three different frequencies ranges of tone threshold audiometry in 102 patients with American Tegumentary Leishmaniasis treated with meglumine antimoniate. Evandro Chagas National Institute of Infectious Diseases, Oswaldo Cruz Foundation, Rio de Janeiro, Brazil.

FR	Category	Sensitivity % (CI)	Specificity % (CI)	PPV % (CI)	NPV % (CI)	FP % (CI)	FN % (CI)
0.25–16 KHz	Total	23 (12–36)	100 (54–100)	100 (74–100)	13(56–26)	0 (0–46)	77 (64–88)
	Up to 59 years	20 (0–35)	85 (70–94)	57 (29–82)	51 (38–63)	15 (6–30)	80 (65–91)
	≥ 60 years	33 (10–65)	100 (66–100)	100 (40–100)	53 (28–77)	0 (0–34)	67 (35–90)
0.25–16 KHz	Total	27 (12–48)	100 (48–100)	100 (59–100)	21 (7–42)	0 (0–52)	73 (52–88)
	Up to 59 years	22 (6–48)	83 (61–95)	50 (16–84)	58 (39–75)	17 (5–39)	78 (52–94)
	≥ 60 years	38 (9–76)	75 (19–99)	75 (19–99)	38 (9–76)	25 (1–81)	62 (24–91)
0.5, 1, 2, 4 KHz	Total	22 (6–48)	100 (63–100)	100 (40–100)	36 (17–59)	0 (0–37)	78 (52–94)
	Up to 59 years	27 (8–55)	85 (65–96)	50 (16–84)	67 (48–82)	15 (4–35)	73 (45–92)
	≥ 60 years	0 (0–71)	56 (21–86)	0 (0–60)	62 (24–91)	44(14–79)	1 (29–100)

FR = frequencies range; PPV = positive predictive value; NPV = negative predictive value; FP = false positive; FN = false negative.

**Table 3:** 3 Sensitivity, specificity, predictive values, false positives and false negatives in the diagnosis of cochlear toxicity from self-reported hearing loss and tinnitus in relation to the ototoxicity alteration at three different frequencies ranges of tone threshold audiometry in 102 patients with American Tegumentary Leishmaniasis treated with meglumine antimoniate. Evandro Chagas National Institute of Infectious Diseases, Oswaldo Cruz Foundation, Rio de Janeiro, Brazil.

FR	Category	Sensitivity % (CI)	Specificity % (CI)	PPV %(CI)	NPV %(CI)	FP %(CI)	FN %(CI)
0.25–16 KHz	Total	28 (17–42)	84 (70–93)	65 (43–84)	52 (40–63)	16 (7–30)	72 (58–83)
	Up to 59 years	22 (11–38)	82 (67–93)	56 (30–80)	51 (38–63)	18 (7–33)	78 (62–89)
	≥ 60 years	50 (21–79)	89 (52–100)	86 (42–100)	57 (29–82)	11 (0–48)	50 (21–79)
0.25–16 KHz	Total	35 (17–56)	78 (58–91)	60 (32–84)	55 (38–71)	22 (9–42)	65 (44–83)
	Up to 59 years	28 (10–53)	83 (61–95)	56 (21–86)	59 (41–76)	17 (5–39)	72 (47–90)
	≥ 60 years	50 (16–84)	50 (7–93)	67 (22–96)	33 (4–78)	50 (7–93)	50 (16–84)
0.5, 1, 2, 4 KHz	Total	33 (13–59)	74 (57–88)	40 (16–68)	68 (51–82)	26 (12–43)	67 (41–87)
	Up to 59 years	33 (12–62)	85 (65–96)	56 (21–86)	69 (50–84)	15 (4–35)	67 (38–88)
	≥ 60 years	33 (1–91)	44 (14–79)	17 (0–64)	67 (22–96)	56 (21–86)	67 (9–99)

FR = frequencies range; PPV = positive predictive value; NPV = negative predictive value; FP = false positive; FN = false negative.

## Discussion

When validating hearing complaints in comparison with audiometry for the diagnosis of ototoxicity, we observed that tinnitus evaluated isolated has greater specificity and positive predictive value in relation to all three frequencies ranges researched, with a low number of false positives, but with a high false negative value.

We observed a higher frequency of CL cases in relation to ML, which is already described in the literature [11]. The highest frequency of ML among the cases treated at our service when compared with the frequency of ML in Brazil has been previously described [11]. Likewise, we observed a higher frequency of men in relation to women, which is in accordance with the American Tegumentary Leishmaniasis endemic in Brazil [1].

Cochlear toxicity was confirmed by pure-tone audiometry and high-frequency audiometry in 53 (52%) patients. We found in the literature several medications with the potential to cause ototoxicity [12], including aminoglycoside antibiotics, which can result in hearing loss. Recent studies indicate an incidence of cochlear alterations of 89% and 93% in weeks 2 and 4 post-treatment with tobramycin [13]. Cisplatin has an incidence in 40%∼97% of children and 20%∼60% of adults [14], while loop diuretics, antibiotics and antimalarials are also associated with this adverse effect [15].

Our findings showed that ototoxicity was associated with MA treatment with 20 mg Sb^5+^/kg/day and with IM treatment. Cochlear toxicity due to MA was previously associated with high dose (20mg Sb^+5^/kg/day), especially in females and ML patients [16]. Likewise, hearing loss resulting from the use of cisplatin and vancomycin has been considered dose-dependent, being associated with high doses of these drugs [17,18].

During auditory monitoring, tinnitus was more frequently self-reported than hearing loss. The complaints of hearing loss and/or tinnitus, which we consider as a sign of clinical toxicity, are already described in cochlear toxicity [19]. Since the perception of the presence of tinnitus is easier than that of hearing loss, tinnitus symptom is frequently reported by the patient before any perception of aminoglycoside-induced hearing loss [20].

The analysis of sensitivity and specificity of self-reported hearing complaints with MA showed lower sensitivity values than specificity values in all evaluated categories. A previous study found that regarding the question “hears well” the sensitivity and specificity values were very low (41% and 31% respectively), so this specific question did not allow the professional to discriminate between who had or didn’t have hearing loss after evaluating the answer [21]. Nonetheless, in another recent study with the use of chloroquine and hydroxychloroquine, the self-report of hearing loss and tinnitus was considered a useful tool for referral to a hearing assessment [22].

The study carried out has limitations regarding the sample size due to the reduction of cases registered in the last five years in Rio de Janeiro, with the lowest number of cases registered in the entire period analyzed being in 2015 [23]. Besides, as the complaints of hypoacusis and tinnitus are subjective, there may have been a misinterpretation of their perception by the patients, which is not possible to measure. Likewise, it is possible that in services without a sector specialized in audiology, this questioning by the health team not be done properly, leading to an over- or underestimation of the perception of these symptoms. Thus, studies with other objective methods that are easier to perform than TTA, such as otoacoustic emissions, are recommended.

ATL is a neglected disease, distributed among the poorest segments of the population [24], with limited access to health care systems and hence to serial audiometric evaluation during treatment. Self-reported hearing loss and tinnitus could be a simple method in the primary health units to raise the suspicion of moderate to severe ototoxicity, requiring intervention in patients under treatment with MA. Although the high specificity could suggest that the absence of hearing complaints could rule out the presence of ototoxicity, making self-report a useful and easy-to-apply tool as screening test for not referring the patient to an audiometry, the high value of false negatives suggests the opposite. Therefore, it would be recommended that audiometric evaluation be performed for patients treated with MA when treated via IM. We acknowledge the great challenge that this recommendation represents, due to the difficulty of resources like an appropriate room with audiological cabin and the availability of specialized professionals. Nonetheless, the low number of false positives suggests the need to value the patient’s complaint for referral. Therefore, the anamnesis with specific questions related to hearing complaints, more specifically tinnitus, might be a useful instrument to direct the patient to audiological evaluations, especially in those patients treated with 20 mg Sb^5+^/kg/day via IM.

## Supporting information

S1 File (SAV)
